# New Challenges and Frontiers in the Research for Neuropsychiatric Disorders

**DOI:** 10.3389/fpsyt.2012.00069

**Published:** 2012-07-16

**Authors:** Daniela Tropea

**Affiliations:** ^1^Neuropsychiatric Genetics Research Group, Trinity College Dublin, St James HospitalDublin, Ireland

Research in neuropsychiatric conditions is living a unique time: genomic analysis advanced the knowledge of the genetic causes of these disorders. At the same time technological advances make possible a deep investigation of the structure and function of the brain (Mothersill et al., 2012) and Induced Pluripotent Stem Cells (IPSC) technology gives us access to neuronal cultures from each single patient (Cheung et al., 2012). These progresses hold the promise for a better understanding of neuropsychiatric disorders with a consequent improvement in the tools for diagnosis and treatment.

Nonetheless, the more we deepen our understanding, the more the complexity appears, and this is due to several reasons: first, except for rare monogenic disorders, the majority of neuropsychiatric conditions are polygenic. Second, the molecular etiology is complex: many of the same susceptibility genes and molecular pathways are emerging across hitherto unrelated disorders – such as autism and schizophrenia – challenging how we conceptualize these conditions (Mitchell, 2011). Third, genetic background interacts with environmental factors to produce the onset and progression of the disease (Stolp et al., 2012) with the consequence that genetic analysis alone cannot predict the outcome of specific clinical symptoms, and environmental factors are critical.

Despite recent advances in genetics and imaging, the neurobiology of neuropsychiatric disorders remains largely obscure. Much of the evidence from genomics implicates molecular mechanisms of neurosynaptic development, plasticity, and function (Mitchell, 2011), and research is converging on deficits in the function of neuronal circuitry. As a consequence, we need to establish additional criteria for the classification of the genes and related phenotypes and new parameters should be taken into account such as the genetics, the neurobiology of the gene products, and the molecular and physiological mechanisms that they control. This task requires a combination of approaches, including animal models and human studies.

In autism research, several steps have been undertaken in this direction and in some cases it has been possible to clarify the intracellular pathways and to produce novel therapeutics (Banerjee et al., 2012). This has been possible for monogenic disorders such as Rett syndrome, Angelmann syndrome, and Fragile X, thanks to the genesis of animal models that present similar genetics and symptoms of the patients. However, this is just a minority of disorders as autism has a complex genetic background. Also for psychosis, research is slowly progressing toward the comprehension of the neurobiology of high penetrant variants in schizophrenia (Kamiya et al., 2012).

To foster new ideas in the neurobiology of neuropsychiatric disorders, it is important to explore new hypothesis regarding the mechanisms of action of the candidate genes and to use appropriate substrates to test them. The use of animal models – even considering the genetic variability due to background and species – is critical to access the developmental brain, and to study the onset and progression of the disorders. Even if the clinical investigation in humans cannot be performed on the rodent models, there are parallel signs that can be used as endophenotypes, such as the reduction in the number of dendritic spines, deficits in working memory, and the structural and functional organization of the brain (Mothersill et al., 2012). Other clues can be found in brain areas rarely explored in neuropsychiatric disorders, such as the visual cortex. Recent evidence proved that synaptic reinforcement in evoked visual stimulation is hampered in patients with schizophrenia (Cavuş et al., 2012). This is not surprising, considering that the majority of candidate genes for psychosis relate to the function of synapses, and that the candidate genes are mutated in all brain areas. Therefore, the visual cortex can be a valid model for investigating the role of candidate genes in circuitry function and plasticity both *in vitro* and *in vivo*, as shown in animal models of autism (Banerjee et al., 2012). Together with the exploration of new brain areas, new players should be taken into account, such as inhibitory circuitry (Chattopadhyaya and Di Cristo, 2012) and microRNAs (Mellios and Sur, 2012). Finally, because several genes are mutant in different conditions, we need to understand the overlapping developmental processes common to several disorders (Bozzi et al., 2012).

In conclusion, the deep understanding of neuropsychiatric disorders requires a combination of techniques, including genetic studies, animal models and imaging, and more than that, requires going beyond the hypothesis currently tested, and explore new mechanisms and endophenotypes. Only using this new, multidisciplinary approach it will be possible to clarify the mechanisms involved in the onset and progression of neuropsychiatric disorders with the perspective of revealing the biological signs of the disorders and identifying new therapeutics.
